# Protective factors against tobacco and alcohol use among pregnant women from a tribal nation in the Central United States

**DOI:** 10.1371/journal.pone.0243924

**Published:** 2021-02-11

**Authors:** Mariah Jorda, Bradley J. Conant, Anne Sandstrom, Marilyn G. Klug, Jyoti Angal, Larry Burd

**Affiliations:** 1 Department of Pediatrics, University of North Dakota School of Medicine and Health Sciences, Grand Forks, ND, United States of America; 2 Department of Population Health, University of North Dakota School of Medicine and Health Sciences, Grand Forks, ND, United States of America; 3 Department of Clinical Research, Alvera Research Institute, Sioux Falls, SD, United States of America; Wayne State University, UNITED STATES

## Abstract

Identifying social determinants of tobacco and alcohol use during pregnancy is critical to improving health outcomes for the next generation. This is especially important on a rural Tribal Nation where influences such as isolation, cultural barriers, and historical trauma have made it uniquely challenging to prevent substance use during pregnancy. The purpose of this study is to identify population-specific factors that are protective against smoking and drinking during pregnancy. We used data from 421 pregnancies collected as a part of the Safe Passages study from a rural Tribal Nation in the central United States. Pregnant women were classified as women who did not smoke (n = 84), women who quit during pregnancy (n = 23), women who smoked during pregnancy (n = 314), and women who both smoked and drank alcohol during pregnancy (n = 149). Demographic data revealed that 28.8% of the mothers were currently employed, and 91.8% of mothers reported a household income of less than $3,000 per year. Substance use rates were higher than national averages: 74.6% smoked during pregnancy and 35.4% of the women both smoked and drank alcohol during pregnancy. Five factors were identified as being protective against substance use during pregnancy: 1) living with someone (81% less likely to smoke and 92% less likely to smoke and drink), 2) having at least 12 years of education (128% less likely to smoke, and 126% less likely to smoke and drink), 3) having over 12 years of education (235% less likely to smoke, and 206% less likely to smoke and drink), 4) being employed (158% less likely to smoke, and 111% less likely to smoke and drink), and 5) not being depressed (214% less likely to smoke, and 229% less likely to smoke and drink). These social determinants should be considered for intervention research to decrease rates of substance use during pregnancy.

## Introduction

Substance use during pregnancy is a major public health concern. Prenatal exposure to tobacco and alcohol can cause detrimental effects which persist throughout the offspring’s lifetime. Smoking during pregnancy is the most common preventable cause of infant morbidity and mortality [1]. Smoking during pregnancy increases the risk of placental abruption, placenta previa, fetal growth restriction, and fetal loss [2, 3]. Maternal cigarette use is significantly associated with preterm deliveries, term low birth weight deliveries, and preterm-related deaths [2]. Up to 34% of sudden unexpected deaths in infants are attributed to prenatal smoking [2]. Some of these deaths are preventable by eliminating smoking during pregnancy. Exposure to tobacco in utero is also associated with birth defects involving the mouth/face, heart, limbs, and gastrointestinal systems [3]. Prenatal tobacco exposure has long-term consequences as well. It has been shown to increase the risk of multisystem effects including childhood asthma [4], recurrent otitis media [5], obesity [6], ADHD [7], and future nicotine addiction [8].

Smoking during pregnancy can compound the effects of prenatal alcohol exposure. Alcohol is a teratogen that freely crosses the maternal-placental barrier and remains in the fetal circulation for an extended period. Maternal alcohol use during pregnancy can cause fetal alcohol spectrum disorders (FASD) characterized by growth retardation, facial deformity, and central nervous system abnormalities [9]. FASD is the leading preventable cause of mental disability [10]. In North Dakota, the prevalence rate of FASD is about 1% of live births [11], although the true rate is certainly higher because the majority of FASD cases remain undiagnosed. Substance use during pregnancy is also an indicator of the mother’s health status. In North Dakota, mothers of children with an FASD diagnosis have a 44.82 fold increase in mortality risk [12].

Although the health risks of substance use during pregnancy are well known, the prevalence is alarming. In the United States, approximately one in five women smoke before pregnancy [13]). These rates are substantially higher in American Indian (AI) populations, where 55.1% of women report smoking during the three months before pregnancy [13]. Although some women are able to successfully quit smoking when they discover the pregnancy, many continue smoking. In the United States, 14.3% of women smoke during pregnancy [13]. The highest prevalence of smoking during pregnancy was reported among American Indian/Alaska Native people with a rate of 26.0% [13]. It has been suggested that Indigenous women in high-income countries “face a disproportionate burden of socioeconomic deprivation and access to culturally appropriate tobacco cessation interventions” [1]. A similar trend is seen in alcohol use during pregnancy. In the United States, it is estimated that 15% of pregnant women consume alcohol and 3% engage in binge drinking [14]. These rates are substantially higher in AI populations of the United States. Approximately 43% of AI women in the United States consume alcohol during pregnancy, and 15% engage in binge drinking [14]. The infant mortality rate from 2010 to 2012 for infants born to AI women was 3.5 times higher than whites [15], and much of the increased mortality may be associated with increased rates of prenatal substance exposure.

These health disparities among AI people must be viewed in the context of a history of genocide, boarding schools, and broken treaties. A study measuring historical trauma among AI found a significant portion of Indigenous adolescents and adults think about historical loss daily or more, and these perceptions are associated with emotional distress and depressive symptoms [16]. This emotional burden, coupled with poverty, discrimination, and other factors, has created conditions permissive of substance abuse in the population. Although substance use rates are a major concern in many Tribal Nations, it is important to recognize that the AI population also has high rates of abstinence. For example, a recent survey indicated that the majority (59.9%) of AI individuals abstain from alcohol, whereas only 43.1% of white individuals abstain from alcohol [17].

Each Tribal Nation has a distinct creation story, history, tradition, and socioeconomic condition and should be considered as such. The Indian Health Service delineates service units to divide the country. One of these service units is the Great Plains Indian Health Service. A recent study from the Indian Health Service reported that out of the 12 regions in the United States, the Great Plains Indian Health Service Area has the highest mortality rates when adjusted for age, the lowest life expectancy at birth (68.1 years), and some of the highest alcohol-related death rates. It is also among the lowest median household incomes and the highest rates of unemployment and poverty [18].

This research was conducted in partnership with the tribe with a goal of developing information for use by the Tribal Nation. The current study will analyze social determinants to identify women at greatest risk for substance use during pregnancy and to determine which social interventions may be effective. The World Health Organization defines social determinants of health as “the conditions in which people are born, grow, live, work, and age” [19]. Differences in these social determinants can produce health inequities, “the unfair and avoidable differences in health status seen within and between countries” [19]. One such health inequity is poor outcomes associated with prenatal exposure to tobacco and alcohol. Several social determinants have been identified as factors correlated with the prevalence of substance use during pregnancy. Most of the research involving substance abuse focuses on risk factors; however, identifying and promoting protective factors is often simpler and more effective. Some protective factors that have been identified against alcohol use during pregnancy include maternal employment [20], being married [20], and in the 2^nd^ or 3^rd^ trimester of pregnancy [20]. Risk factors for alcohol use include depression [21, 22], anxiety [21], being unmarried [22], belonging to a lower socioeconomic status [22], and being black [22] or white [20]. Protective factors against smoking during pregnancy include attending one prenatal consult [23], more years of schooling [23], living with a partner [21, 23], having no previous children [23], and the absence of alcohol use [23]. Having PTSD [24] or depression [21, 25] increases the risk of both alcohol and tobacco use during pregnancy.

None of these studies focused on Tribal Nations, where culturally appropriate screening and interventions are desperately needed. Previous researchers have called for population specific studies to direct resources and policy. The purpose of this study is to examine the demographic information of pregnant women from one Tribal Nation and to identify any characteristics that are protective against prenatal substance use.

## Methods

A waiver of participant consent was approved by the University of North Dakota Institutional Review Board. Access to the data was approved by the tribal council.

### Enrollment

Data were collected in the Safe Passage Study conducted by the Prenatal Alcohol in Sudden Infant Death Syndrome (SIDS) and Stillbirth (PASS) Network. The full methodology of the study can be found in Dukes et. al., 2014 [26]. Screening and enrollment occurred at a clinical site on a Tribal Nation. Consenting women were enrolled between 6 weeks gestation up to delivery. From the Safe Passage Study, “A woman is eligible if all of the following criteria are met: (1) able to provide informed consent, (2) pregnant, (3) 16 years of age or older, and (4) gestational age of at least 6 weeks, 0 days and not at the delivery admission. A woman is excluded if any of the following criteria are met: (1) planned abortion, (2) planned relocation from catchment area prior to delivery, or (3) advice against participation by a health care provider (e.g. requires additional medical care)” [26].

In a published study the Safe Passage group after adjustment for demographic and clinical characteristics demonstrated an increase in relative risk for SIDS of 11.97 which was highly significant (95% CI: 2·59–53·7, *p* < 0·001) in infants whose mothers reported both prenatal drinking and smoking beyond the first trimester [27]. For drinking only after the first trimester and for smoking only beyond the first trimester a significant increase in risk was not detected when compared to those unexposed or women who reported quitting early in pregnancy [27]. Another study by the group demonstrated that even low levels of exposure to prenatal alcohol or smoking were associated with changes in brain development [28]. Prenatal alcohol and cigarette exposure also appear to impact newborn cardiac physiology [29].

### Factors studied

A previous study analyzed congregate data from the Safe Passage Study including white and AI women in the Great Plains Indian Health Service Northern Plains of the US, and women of mixed ancestry in South Africa [30]. Group-based trajectories were used. Women who smoked and drank continuously through pregnancy were more likely to have enrolled in the study later in pregnancy, have less than 12 years of education, be of mixed ancestry, and be depressed than those categorized in the none/minimal or quit groups. The purpose of this current study is to look specifically at one distinct Tribal Nation in the Great Plains Area to determine if the same exposure relationships applied. This study is unique in that it does not use group-based trajectories as the sample size is much smaller.

In addition to the mother’s education and depression classification, the current study looks at other factors which have been studied in relation to prenatal substance use: maternal living arrangement (alone or with someone) [21, 23], yearly income [22], and PTSD classification [24]. It also investigates if the education or employment of the father and mother together would alter maternal smoking rates. Resilience is the ability to resist stress and return to normal homeostasis state [30]. Previous studies have shown resilience to be inversely related to substance use [31], so this study examines resiliency classification as a protective factor that might offset the difficult conditions on this Tribal Nation. Protective factors were chosen for analysis as they can be a focus of future health promotion activities to counteract risk factors and decrease substance use.

### Maternal interviews

Women were interviewed at up to three prenatal visits at 20–24 weeks, 28–32 weeks, and 34+ weeks. At the first prenatal visit, maternal demographic characteristics were self-reported including age, race, education, and partner status. Behavioral risk factors of the mother were obtained using medical record abstraction and through the administration of several surveys including the Edinburgh Postnatal Depression Scale (positive was defined as greater than 13), Harvard Trauma Questionnaire (high PTSD was defined as a score of 45 or greater), and the Connor Davidson Scale (low resiliency was defined as a score less than 100). In the Safe Passage study, several embedded studies that were site-specific were added over the 13 year study period. These included measures of PTSD and trauma. As a result, the sample sizes for these variables differ from the larger study.

### Prenatal exposure assessment

Alcohol exposure information was collected by maternal self-report using the well validated Timeline Follow-Back method [32]. Reported alcohol intake was standardized to total grams of alcohol consumed and converted into standard drinks (14 g of absolute alcohol). Prenatal smoking exposure information was obtained by asking how often the participant smoked a tobacco cigarette and the number of cigarettes smoked on a typical day.

### Statistical analysis

Women were classified into four groups: 1) women who did not smoke, 2) women who quit during pregnancy, 3) women who smoked during pregnancy, and 4) women who both smoked and drank alcohol during pregnancy. Demographic risk factors were categorized as living alone (single, partnered living alone, divorced, widowed) or with someone (married, partnered living together), mother’s education in years, employment of the mother and the partner in the past year, and mother’s yearly income. Maternal behavioral risk factors that were analyzed were feelings of depression, resiliency score, and post-traumatic stress disorder (PTSD) score.

Chi-square tests were used to find associations between the risk factors and three levels of smoking: quit smoking, smoked during pregnancy, and both smoked and drank during pregnancy. Women who did not smoke were compared to the three levels of smoking. Relative risks (RRs) were estimated for the binary risks to find protective factors for not smoking versus the any smoking and combined smoking and drinking. The confidence intervals are at the 95^th^ percentile. Quit smoking was not used as a separate group comparable to not-smoking due to small number of women who quit. Two logistic regressions were run with living status, mother’s years of education, mother’s unemployment, and depression as predictors of the smoking levels. Resiliency and PTSD were not used in the logistic regressions due to the low number of mothers being measured for these variables.

## Results

There were 421 births in the data set between August 2007 to January 2015. As the birth rate is about 125 births per year on this Tribal Nation, our sample of 421 of 916 births (44.9%) was substantial. The average age of the mother was 24.7 (S.D. 0.3, range 16 to 43).

Approximately half (47.0%) of the women lived alone, and 52.9% lived with a partner. 47.7% of the women completed 6–11 years of education, 27.8% completed 12 years of education, and 24.5% had more than 12 years of education. A similar trend is seen with the partner’s education, with both partners completing high school in 41.6% of couples.

The unemployment rate is elevated in this Tribal Nation. Among study participants, 33.3% of the mothers had been unemployed for over a year, and 28.8% of the mothers were currently employed. In 39.7% of the couples, neither parent was employed. The 2020 poverty guideline set by the U.S. Department of Health and Human Services for a 3 person household is $21,720 [33]. In this Tribal Nation, 91.8% of mothers reported a household income of less than $3,000 per year. 18.7% of women reported a household income of less than $500 per year.

**Fig 1:**
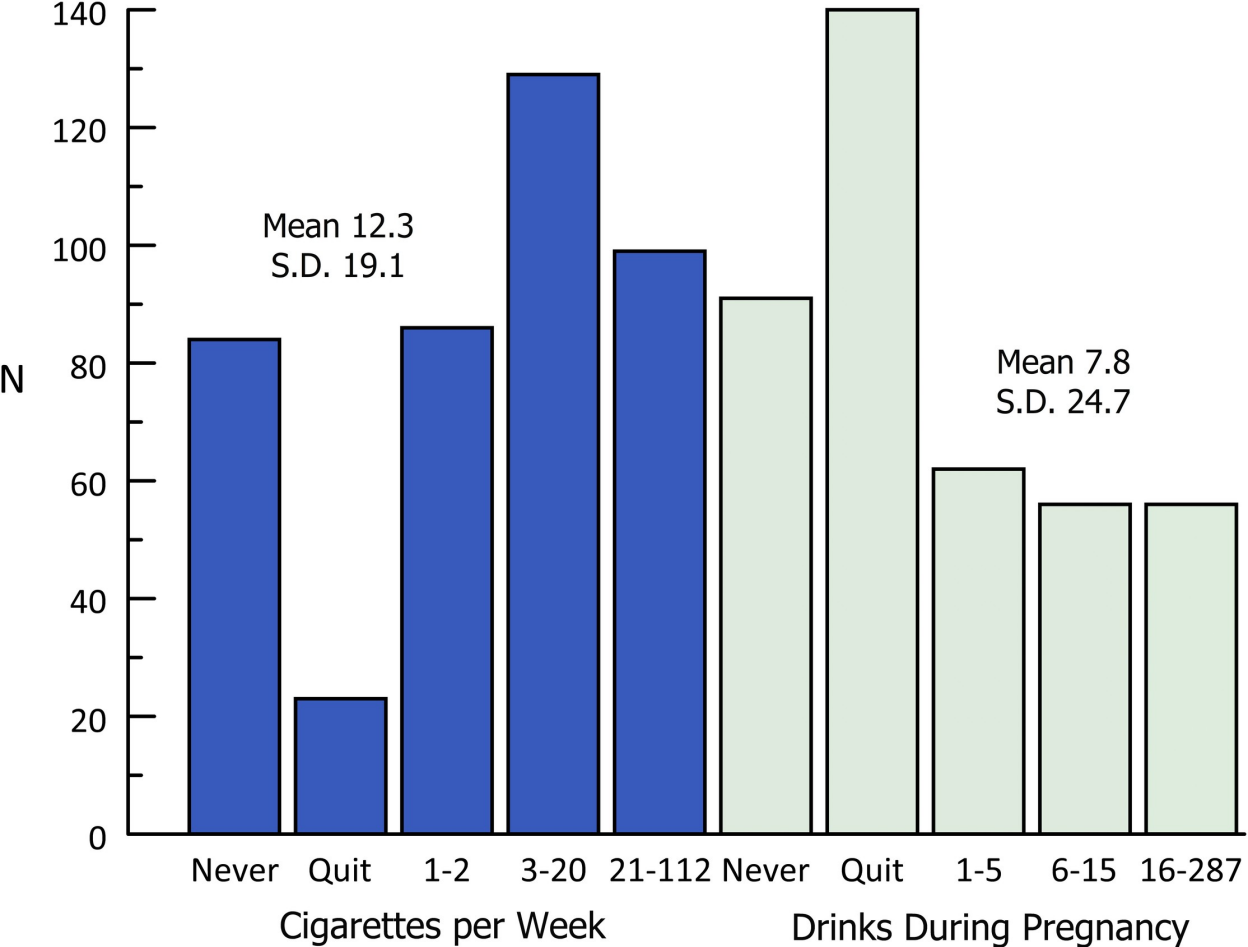
Prenatal exposure to alcohol and cigarettes. The distribution of cigarettes per week and the drinks during pregnancy.

Assessment of smoking found that 19.9% of mothers did not smoke, 5.5% of the mothers quit smoking during pregnancy and 74.6% smoked during pregnancy. Those who smoked averaged 16.5 (S.D. 1.16, range 0.02 to 112) cigarettes per week. In Fig 1, we present the distribution of smoking and drinking. Many women quit smoking and drinking when pregnant. This is likely due to the cost of cigarettes and that they smoke mostly when they drink. Also, many women do not smoke in their house if they have children. In this sample, 35.4% of the women both smoked and drank alcohol during pregnancy. In the study period, 40% of the women drank during pregnancy, 34% quit drinking during pregnancy, and 2% drank continuously during pregnancy. The rates of drug use during pregnancy was very low. Table 1 shows the possible maternal protective factors for not smoking. Some associations were found between those who quit smoking when they learned of their pregnancies and those who never smoked, including whether the mother is living with someone, her education level, and her income level. Mothers who lived with someone were more likely to not smoke (25% to 14%) than those who quit smoking (4% to 7%), smoked (70% to 79%), or both smoked and drank (30% to 42%) (all p < .001). Completing high school or a higher level of education was also associated with not smoking. Being employed at least part time during the past year was associated with not smoking relative to both women who smoked and those who smoked and drank. Not having depression and PTSD were also associated with not smoking, though resiliency was not. Depression was a finding in the medical record review for 18.7% of the mothers. Clinically diagnosed depression was associated with an increased risk of smoking, drinking, or both during pregnancy, but depression as assessed on the Edinburgh Scale was not associated with an increased risk.

**Table 1:** Prevalence of smoking and drinking during pregnancy associated with mother’s demographics.

	Total	Did not smoke	Quit	Smoked	Smoked & Drank
	N	N (%)	N (%)	N (%)	N (%)
	421	84 (19.9%)	23 (5.5%)	314 (74.6%)	149 (35.4%)
**Living:**	N = 421				
Alone	198 (47.0%)	28 (14.1%)	13 *(6.6%)	157 *(79.3%)	83 *(41.9%)
With Someone	223 (52.9%)	56 (25.1%)	10 (4.5%)	157 (70.4%)	66 (29.6%)
**Mother’s Education:**	N = 421				
6–11 Years	201 (47.7%)	25 (12.4%)	14 *(6.9%)	162 *(80.6%)	76 *(37.8%)
12 Years	117 (27.8%)	28 (23.9%)	3 (2.6%)	86 (73.5%)	40 (34.2%)
More Than 12 Years	103 (24.5%)	31 (30.1%)	6 (5.8%)	66 (64.1%)	33 (32.0%)
**Parents’ Education:**	N = 320				
Neither Completed HS	53 (16.6%)	10 (18.9%)	1 *(1.9%)	42 *(79.3%)	22 (41.5%)
Mother Completed HS	43 (13.4%)	11 (25.6%)	3 (6.9%)	29 (67.4%)	15 (34.9%)
Partner Completed HS	91 (28.4%)	10 (10.9%)	11 (12.1%)	70 (76.9%)	29 (31.9%)
Both Completed HS	133 (41.6%)	41 (30.8%)	4 (3.0%)	88 (66.2%)	42 (31.6%)
**Unemployed > 1 Year:**	N = 421				
Yes	140 (33.3%)	17 (12.1%)	6 (4.3%)	117 *(83.6%)	50 *(35.7%)
No	281 (66.8%)	67 (23.8%)	17 (6.1%)	197 (70.1%)	99 (35.2%)
**Parents’ Employment:**	N = 320				
Neither Employed	127 (39.7%)	13 (10.2%)	8 (6.3%)	106 *(83.5%)	46 *(36.2%)
Mother Employed	39 (12.2%)	15 (38.5%)	3 (7.7%)	21 (53.9%)	10 (25.6%)
Partner Employed	101 (31.6%)	23 (22.8%)	5 (4.9%)	73 (72.3%)	32 (31.7%)
Both Employed	53 (16.6%)	21 (39.6%)	3 (5.7%)	29 (54.7%)	20 (37.7%)
**Yearly Income:**	N = 379				
< = $500	71 (18.7%)	12 (16.9%)	5 *(7.04%)	54 *(76.1%)	27 *(38.0%)
$500 - $1,000	125 (32.9%)	26 (20.8%)	11 (8.8%)	88 (70.4%)	35 (28.0%)
$1,001 - $2,000	104 (28.2%)	21 (20.2%)	2 (1.9%)	81 (77.9%)	42 (40.4%)
$2,001 - $3,000	45 (11.9%)	4 (8.9%)	4 (8.9%)	37 (82.2%)	22 (48.9%)
> $3,000	34 (8.9%)	17 (50.0%)	1 (2.9%)	16 (47.1%)	9 (26.5%)
**Depressed:**	N = 379				
Yes	71 (18.7%)	8 (11.3%)	3 (4.2%)	60 *(84.5%)	33 *(46.5%)
No	308 (81.3%)	72 (23.4%)	20 (6.5%)	216 (70.1%)	102 (33.1%)
**Resiliency:**					
Low (<100)	52	12 (23.1%)	2 (3.8%)	38 (73.1%)	20 (38.5%)
High (> = 100)	61	17 (27.9%)	5 (8.2%)	39 (63.9%)	17 (27.9%)
**PTSD:**					
High (> = 45)	17	1 (5.9%)	1 (5.9%)	15 *(88.2%)	9 *(52.9%)
Low (<45)	28	13 (46.4%)	1 (3.6%)	14 (50.0%)	5 (17.9%)

No smoking at all during pregnancy compared to quit smoking, smoked during pregnancy, and both smoked and drank during pregnancy.

* p < .05

**Fig 2:**
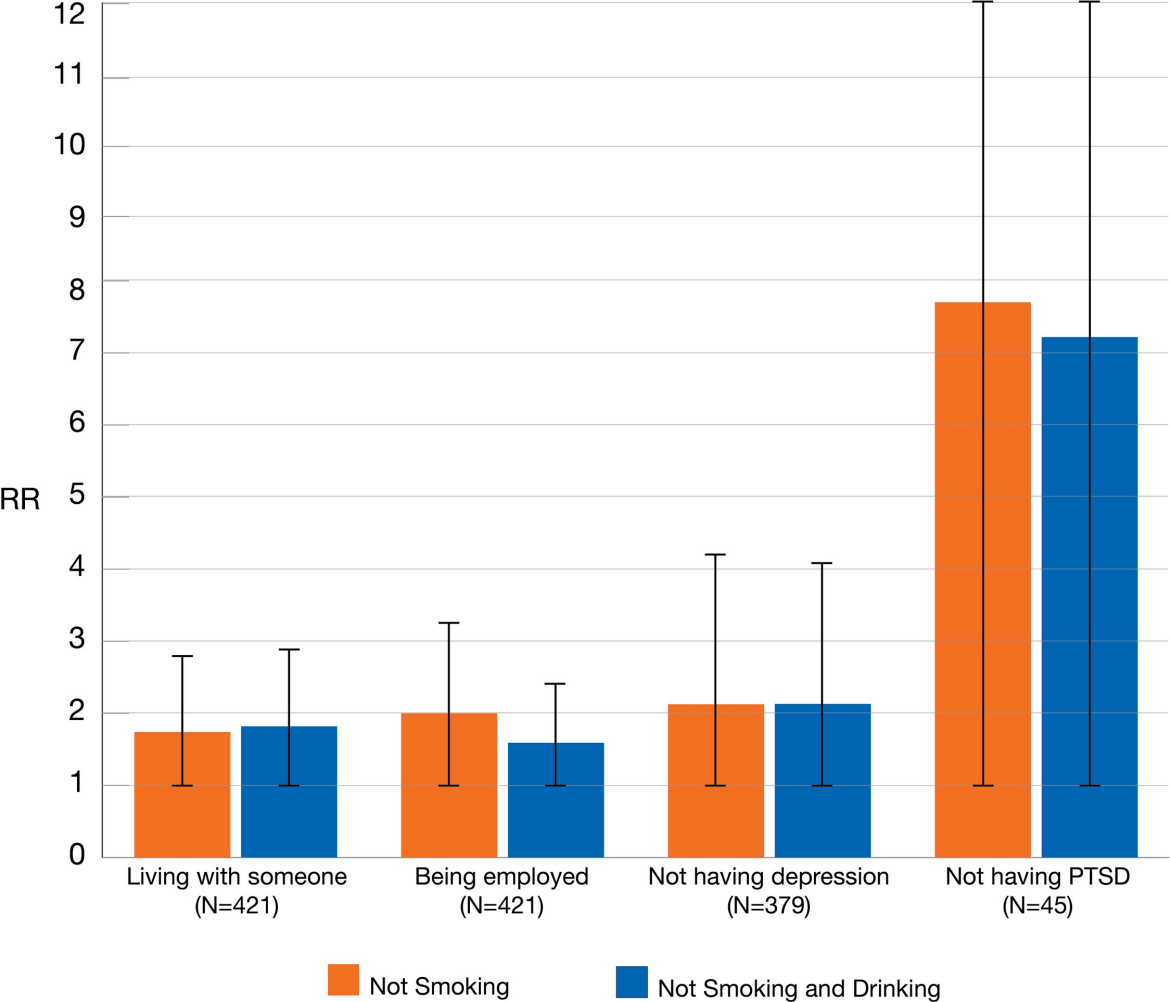
Likelihood of not smoking or not both smoking and drinking during pregnancy. This figure shows the relative risk of 1) not smoking during pregnancy and 2) not both smoking and drinking during pregnancy. These risks are based on maternal factors: living with someone, being employed, not having depression, and not having PTSD. They are all statistically significant. Each factor is protective against smoking during pregnancy and both smoking and drinking during pregnancy. Being employed is a stronger factor against only smoking than both smoking and drinking. Not having PTSD is the strongest protective factor, but also has the largest error due to small sample size. The upper limits of the CI’s for PTSD are not shown- they are 53.5 and 47.4.

The protective risk of not smoking from smoking or both smoking and drinking given maternal attributes are shown in Fig 2. The relative risk (RR) of not smoking compared to any smoking is presented in the blue bar; the RR compared to smoking and drinking is also shown with the orange bar with 95^th^ percentile limits. Not living alone is a similar protective factor for not smoking when compared to both smoking groups. The RRs were 1.7 (CI = 1.2–2.6) and 1.8 (CI = 1.3–2.6) respectively. Not having a record of depression in the medical record was also a very similar protective factor for both smoking groups; RR = 2.1 (CI = 1.1–4.2) and RR = 2.1 (CI = 1.1–4.0) respectively. Employment by the mother had a stronger protective effect against smoking (RR = 2.0, CI = 1.2–3.3) than smoking and drinking (RR = 1.6, CI = 1.0–2.5). Not having PTSD appeared to have the strongest protective factor against smoking (RR = 7.7, CI = 1.1–53.5) and smoking and drinking (RR = 7.2, CI = 1.1–47.4), though the small number of women (N = 45) created the largest error estimate.

Logistic regressions were run to determine if any confounding of mother’s factors affected the strengths of their relationships to not smoking. Mother and father’s education and employment were highly correlated, and only mother’s education (6–11 years reference value) and mother’s employment within the past year were used. Final models of four variables are shown in Table 2.

**Table 2:** Logistic regression for not smoking or not both smoking and drinking.

	Not Smoking (N = 356)		Not Both Smoking and Drinking (N = 215)	
	OR	95% CI	OR	95% CI
Living with someone	1.8	(1.0–3.2)	1.9	(1.0–3.6)
Education 12 years	2.30	(1.2–4.3)	2.3	(1.1–4.6)
Education >12 years	3.4	(1.7–6.5)	3.1	(1.5–6.5)
Being employed	2.6	(1.4–4.7)	2.1	(1.1–4.2)
No record of depression	3.2	(1.4–7.2)	3.3	(1.4–8.0)

Women who live with someone were 81% less likely to smoke, and 92% less likely to smoke and drink. Education showed an increase in likelihood of not smoking as the years of education increased (OR = 2.3 to OR = 3.4, and OR = 2.3 to OR = 3.1). Women with at least 12 years of education were 128% less likely to smoke, and 126% less likely to smoke and drink than women. Women with over 12 years of education were 235% less likely to smoke, and 206% less likely to smoke and drink. Working more than doubled the likelihood of not smoking. Not being diagnosed with depression more than tripled the likelihood of not smoking. Women who were employed were 158% less likely to smoke and 110% less likely to smoke and drink. Women who were not depressed were 214% less likely to smoke and 228% less likely to smoke and drink during pregnancy.

## Discussion

This research reveals a disproportionate rate of poverty and unemployment among pregnant women from this Tribal Nation. Approximately half of the women did not complete high school, under 30% were currently employed, and over 90% reported a household income of less than $3,000 per year. According to the USDA’s 2015 report, it costs approximately $9,330 per year to raise a child for a family in the lower third of the income distribution [34]. Over 90% of the pregnant women in the study make less than one third of the estimated yearly cost of providing for one child. There is a need for improved employment opportunities for families on this rural reservation. While food stamps, Women Infant Child (WIC), Healthy Start, food banks, and tribal support are widely used, these are currently inadequate. Commodities are used but have considerable stigma and are considered as unhealthy.

Disparities in substance use rates among women in this study were also evident. Nearly three quarters of the women smoked during pregnancy. Given previous reports that 14.3% of women smoke during pregnancy in the U.S. [13], pregnant women on this Tribal Nation were over 5 times more likely to smoke. Even compared to American Indian/Alaska Native population data that indicated a smoking rate of 26.0% during pregnancy [13], women on this rural Tribal Nation were 2.9 times more likely to smoke during pregnancy. Alcohol use during pregnancy on this Tribal Nation was also higher than the U.S. average (40% vs. 15% respectively) [14]. However, the rates of drinking during pregnancy reported by the Indian Health Service were slightly lower than the American Indian/Alaska Native population overall (43%) [14].

Additional resources are clearly needed to address the disparities associated with the high rates of substance use during pregnancy on this Tribal Nation. Healthy People 2020 includes three national health objectives related to smoking among women during the perinatal period: reduce the prevalence of smoking before pregnancy to 14%, reduce the prevalence of cigarette smoking among pregnant women to 1.4%, and increase the percentage of pregnant women who stop smoking during pregnancy to 30% [35]. Progress toward achieving these goals requires empowering AI women and promoting the factors that protect against substance use during the prenatal period, such as those identified in this study.

Prioritizing decreasing substance use during pregnancy is motivated by both health risks and cost. The adverse maternal and infant health outcomes due to smoking and drinking during pregnancy carry heavy financial costs. In 2004, it was estimated that approximately $122 million in healthcare costs for infant hospitalization after delivery were attributed to prenatal smoking in the United States [36]. This is $653 per mother who smoked [36]. It is financially worthwhile to spend money on prevention of maternal substance use in order to save money treating infants in the Neonatal Intensive Care Unit (NICU) and to prevent long term health care utilization as a result of chronic conditions.

Pregnancy can bring many changes in a woman’s life and is often an opportune time to encourage lifestyle changes such as smoking cessation. Women who quit smoking during pregnancy are more likely to be abstinent up to 21 years after pregnancy than women who continue to smoke during pregnancy [37]. Pregnant women who smoke should be provided information and offered effective smoking cessation interventions.

There are several intervention options currently available including screening for substance use with a validated screen, counseling, financial incentives, and pharmacotherapy. On this rural Tribal Nation, the local health center pharmacy provides a smoking cessation program that is offered to women during their prenatal visits. An addiction counselor in the behavioral health program is also available to help pregnant women with alcohol or drug use concerns. The results of these preventive programs are likely represented in this study by the 25% of women who never smoked or quit smoking during pregnancy, as well as the 34% of women who quit drinking during pregnancy.

We propose supplementing these programs with population-specific data-based interventions involving the protective social determinants identified in this study. No current intervention has a success rate as great as the social determinants results. For example, women who are not depressed were 228% less likely to smoke and drink during pregnancy. Resources used toward mental health screening and treatment would have the dual effect of promoting positive mental health and decreasing prenatal substance use. Other interventions might include promoting education up to and beyond 12 years, providing employment opportunities, and promoting social support.

The protective social determinant results of this study could also be used to stratify women into high and low risk groups for substance use during pregnancy. Identifying protective factors could have a practical use for healthcare professionals working with this population. For example, a screening tool could be developed that would look for women who are unemployed, have depression, or have less than 12 years of education. By understanding these factors, it is possible to examine the effect of focusing resources toward women who are most at risk for substance use during pregnancy.

This study has several limitations. Since the alcohol and cigarette exposure information was collected through maternal self-report, our data could substantially underestimate substance use due to social stigma and recall error. Therapeutic drift might also be a concern, in which women would have altered their behavior because they were enrolled in the study. There are also some gaps in the data due to women enrolling late in pregnancy or missing assessments. The sample size for some of the behavioral factors such as PTSD rating and resiliency was small, as not all participants were evaluated using those questionnaires. Finally, the goal of this study was to examine population specific conditions on this small, rural Tribal Nation. The sample was uniform, and the results are not generalizable to other populations.

Further research is needed to determine if there are causal relationships between the social determinants and substance use during pregnancy. More information is also needed to develop strategies for implementing changes to social determinants such as providing access to more education, preventing, identifying, and treating depression, increasing employment rates, and promoting familial bonding and social support.

## Conclusion

**Fig 3:**
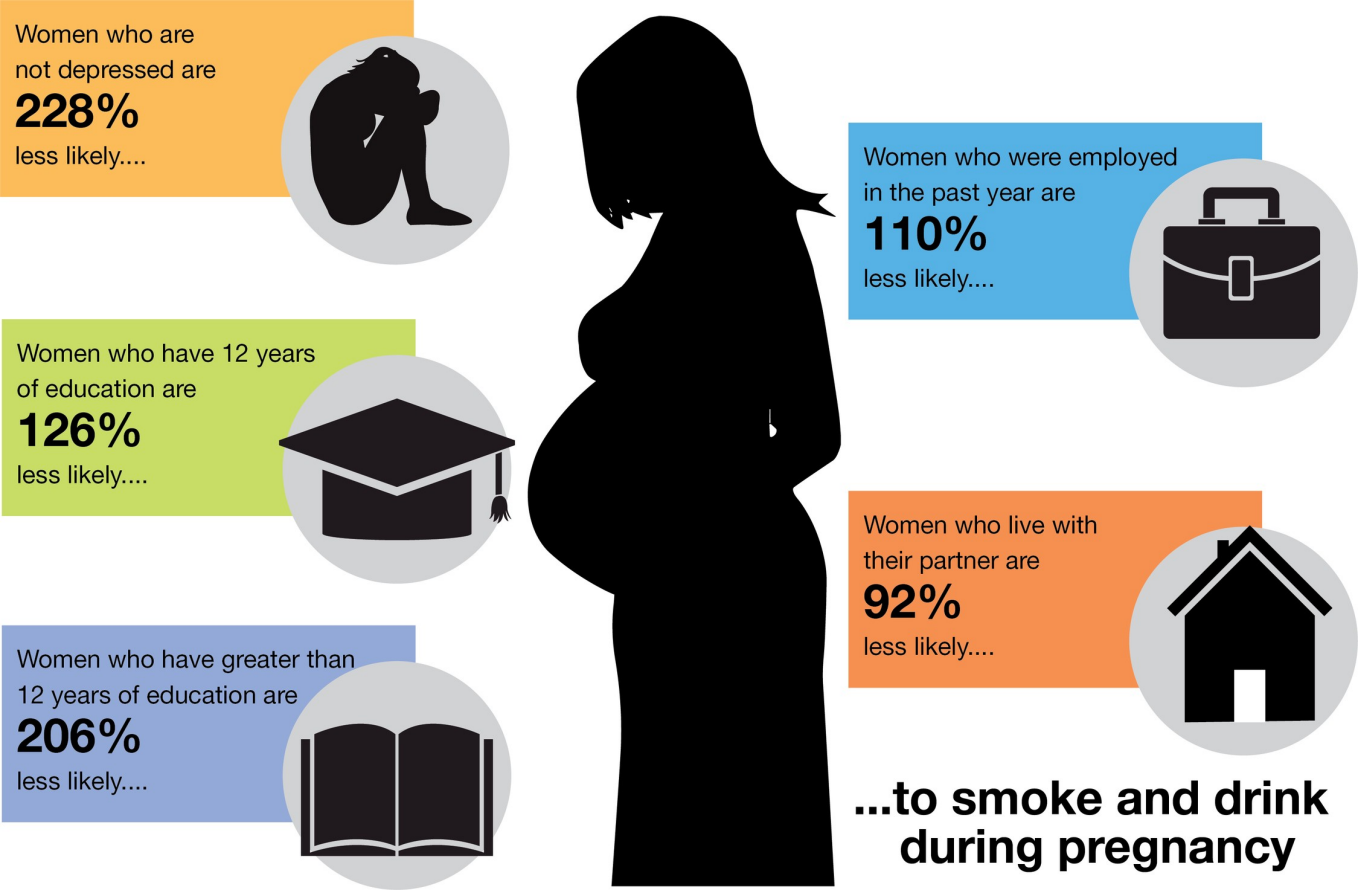
Protective factors–what factors protect against smoking and drinking during pregnancy?. The five maternal factors which were associated with decreased risk for both smoking and drinking during pregnancy.

Smoking during pregnancy is a major cause of preventable morbidity and mortality among infants. AI populations in rural areas face additional challenges in preventing substance use during pregnancy. On one Tribal Nation, approximately three quarters of the women smoked during pregnancy and 35.4% both smoked and drank during pregnancy. These rates are much greater than the United States national average, and the results of substance use during pregnancy will affect this population for generations. In Fig 3 we depict the five factors that are protective against both smoking and drinking during pregnancy: 1) not being depressed (228% less likely), 2) having more than 12 years of education (206% less likely), 3) having at least 12 years of education (126% less likely), 4) being employed in the past year (110% less likely), and 5) living with a partner (92% less likely). These social determinants are modifiable factors that should be the emphasis of future research to decrease substance use during pregnancy.
